# Approaches to dissect the vitamin biosynthetic network of the gut microbiota

**DOI:** 10.20517/mrr.2025.66

**Published:** 2025-10-20

**Authors:** Chiara Tarracchini, Francesca Bottacini, Leonardo Mancabelli, Gabriele Andrea Lugli, Francesca Turroni, Douwe van Sinderen, Marco Ventura, Christian Milani

**Affiliations:** ^1^Laboratory of Probiogenomics, Dept. Chemistry, Life Sciences and Environmental Sustainability, University of Parma, Parma 43124, Italy.; ^2^APC Microbiome Ireland, University College Cork, Cork T12 YN60, Ireland.; ^3^Department of Biological Sciences, Munster Technological University, Cork T12 P928, Ireland.; ^4^Department of Medicine and Surgery, University of Parma, Parma 43124, Italy.; ^5^Microbiome Research Hub, University of Parma, Parma 43124, Italy.; ^6^School of Microbiology, Bioscience Institute, National University of Ireland, Cork T12 Y337, Ireland.

**Keywords:** Microbiome, metagenomics, microbe-microbe interaction

## Abstract

B-group vitamins and vitamin K are essential micronutrients required for numerous cellular processes in both microbial and human physiology. While traditionally considered to originate predominantly from dietary sources, the biosynthetic capacity of the human gut microbiota has recently been recognized as a valuable, though historically underappreciated, endogenous source of these vitamins. In particular, the microbial contribution to the host vitamin pool is increasingly acknowledged as a functionally relevant aspect of vitamin homeostasis, especially in the colon, where microbiota-derived vitamins may be absorbed via specific transport mechanisms. This review provides a comprehensive overview of our current understanding of the biosynthesis of B-group vitamins and vitamin K by human gut-associated bacteria, with particular emphasis on key methodologies employed to assess if, how and to what extent members of the gut microbiota supply their host with such micronutrients. Through an integrated overview of available evidence, we highlight both the progress made and the outstanding challenges in elucidating the microbial contribution to the host vitamin metabolism.

## GENERAL INTRODUCTION

Vitamins are a chemically diverse group of organic compounds that are required in trace amounts yet are indispensable for maintaining cellular and systemic physiology across all domains of life. Unlike macronutrients, which serve as structural components or energy sources, vitamins exert their functions primarily through their roles as cofactors or coenzyme precursors, enabling a wide range of enzymatic reactions involved in central metabolism, biosynthesis, redox regulation, and cellular signaling^[1-4]^. In humans, even marginal vitamin deficiencies can compromise metabolic integrity and increase susceptibility to a wide array of pathological conditions, including anemia, neurodegeneration, and immunodeficiency^[5,6]^.

Based on solubility and physiological behavior, vitamins are traditionally classified into water-soluble and fat-soluble groups. Water-soluble vitamins include the B-complex group [thiamine (B1), riboflavin (B2), niacin (B3), pantothenic acid (B5), pyridoxine (B6), biotin (B7), folate (B9), and cobalamin (B12)] and vitamin C^[7]^. These compounds typically act as prosthetic groups or coenzymes in enzymatic complexes, particularly in energy metabolism, nucleotide synthesis, and one-carbon transfer reactions^[8,9]^. Due to their hydrophilic nature, water-soluble vitamins are not stored efficiently in tissues and must be acquired regularly through the diet.

Fat-soluble vitamins (A, D, E, and K) are absorbed via lipid-mediated pathways and accumulate in adipose tissue and liver, providing a more stable reservoir. While their roles are more regulatory rather than catalytic, they are essential for optimal vision, immune modulation, antioxidant defense, and calcium-phosphate homeostasis^[10]^. Notably, vitamin K serves as a cofactor for the γ-carboxylation of glutamate residues in clotting factors^[11]^.

Remarkably, the inability of humans to synthesize most vitamins makes these micronutrients essential nutrients^[12]^. Only a few exceptions exist, such as vitamin D, which can be made from 7-dehydrocholesterol in the skin under ultraviolet (UV) exposure^[13]^. For all others, including all water-soluble vitamins and vitamin K, humans are dependent on external sources, namely dietary intake and microbial production.

Unlike higher eukaryotes, numerous bacterial species encode the genetic machinery for *de novo* synthesis of essential cofactors, including B1, B2, B3, B5, B6, B7, folate (B9 or B11), B12, and menaquinones (K2)^[14,15]^. Notably, 6 out of these 8 B-group vitamins, B1, B2, B3, B5, B9, and B6, are direct biosynthetic precursors of essential cofactors conserved from bacteria to mammals^[16]^, such as thiamine pyrophosphate (TPP), flavin mononucleotide/flavin adenine dinucleotide (FMN/FAD), nicotinamide adenine dinucleotide (NAD/NADP), coenzyme A (CoA), pyridoxal phosphate (PLP), and tetrahydrofolate (THF), respectively. Biotin serves as an essential carboxylation/decarboxylation cofactor, playing an important role in lipogenesis, carbohydrate and amino acid metabolism^[17]^. Cobalamin, a precursor of the B12 coenzyme family including cyanocobalamin, methylcobalamin, and adenosylcobalamin, is essential for all animals and many, but not all, bacterial species^[18,19]^.

In this context, the biosynthetic capabilities of the human gut microbiota have recently attracted growing attention as a potential endogenous source of B-group vitamins and vitamin K and are increasingly recognized as a functionally relevant contributor to host vitamin homeostasis^[8,20-22]^. This paradigm shift has been driven by the refinement and diversification of methodological approaches, which have enabled the detection, quantification, and functional interpretation of microbial vitamin biosynthesis with increasing resolution^[23]^. As a result, the field has moved beyond descriptive observations toward mechanistic insights, highlighting the pivotal role that methodological innovation has played in advancing the field, thereby facilitating our enhanced appreciation of microbe-host vitamin interactions.

In the following sections of this review, we provide a comprehensive overview of the current understanding of the biosynthesis of B-group vitamins and vitamin K by human gut-associated bacteria. Particular consideration will be devoted to the methodological approaches currently available to investigate the vitamin-producing potential of the gut microbiota, including genomics, metagenomics, transcriptomics, and metabolomics, with the aim of highlighting opportunities and challenges in this emerging field.

## VITAMIN BIOSYNTHESIS IN THE GUT MICROBIOTA

### The gut microbiota as a source of vitamins

The human gut microbiota constitutes a dense and metabolically active microbial ecosystem, estimated to harbor over 10^13^ microbial cells and an immense genetic reservoir of functions, knowledge on which continues to grow as new species and functions are discovered^[24,25]^.

Vitamin biosynthesis by gut microorganisms, particularly the eight water-soluble B-group vitamins, such as thiamine, riboflavin, niacin, pantothenic acid, pyridoxine, biotin, folate, and cobalamin, as well as vitamin menaquinones, has received increasing scientific interest over the past decades, prompted by growing evidence of their potential nutritional relevance to the host^[8,20-23]^. As an illustrative case, germ-free rats maintained on vitamin K-deficient diets exhibit reduced prothrombin levels and are prone to spontaneous hemorrhages. In contrast, conventionally raised counterparts display normal prothrombin levels and unaltered coagulation function, despite receiving the same dietary regimen^[26]^. Considering that dietary supplementation studies in rats indicate that approximately 6-10 µg/g of diet of menaquinone-4 represent the lowest concentration required to restore baseline plasma prothrombin concentrations after one week on a vitamin K-deficient diet^[27]^, it is plausible to speculate that microbially derived menaquinones in the murine colon may reach physiologically relevant levels close to this range, thereby contributing to the maintenance of normal coagulation function.

Similarly, in human studies, a low-vitamin K diet for 3-4 weeks did not lead to measurable vitamin deficiency, unless their gut microbiota was concurrently suppressed by broad-spectrum antibiotic treatment. Under these conditions, a notable decline in plasma prothrombin was observed, clearly underscoring the important gut microbiota-mediated contribution to maintaining vitamin K homeostasis^[28]^.

In parallel with *in vitro* studies, several *in silico* investigations have explored the potential contribution of gut microbiota to host-relevant micronutrient intake^[22,29]^, particularly in relation to the Dietary Reference Intake (DRI) of B vitamins^[29]^. These computational approaches have identified microbial biosynthetic pathways capable of producing various B vitamins, suggesting a potential contribution of the gut microbiota to host micronutrient intake^[29]^. Specifically, although this remains speculative, it has been proposed that the (gut) microbial synthesis of vitamin B3, B6, B9, and B12 may account for approximately 27%-86% of the respective DRI in humans^[29]^. Moreover, the detection of B vitamin transport systems in the human colon has reinforced the notion that microbial and dietary vitamins may act synergistically in maintaining systemic vitamin homeostasis. However, this perspective remains uncertain, as the actual bioavailability of these microbially produced vitamins in the colonic environment is still poorly characterized. Unlike dietary vitamins, whose absorption efficiency in the small intestine is well defined, the uptake of microbially synthesized vitamers depends on their chemical form and the competence of host transporters. For example, microbially synthesized corrinoids, including variants of vitamin B12, are numerous in the colonic lumen and frequently differ structurally from the biologically active cobalamin, resulting in reduced bioavailability for the human host^[19,30]^. Moreover, given that microorganisms synthesize B vitamins for their own metabolism, the extent of surplus, whether actively secreted or released through spontaneous bacterial cell lysis, may represent only a minor fraction of the total input compared to dietary sources^[19,31]^. Consequently, while their contribution to systemic vitamin pools may be limited, microbially derived vitamins are nonetheless widely recognized for their biologically significant role in shaping the local intestinal environment, particularly within the lumen and at the mucosal interface^[32]^. In this context, microbial vitamins are known to support the metabolic needs of colonic epithelial cells (colonocytes), influence barrier integrity, modulate local immune responses, and exert antioxidant and protective roles^[33-36]^.

**Figure 1:**
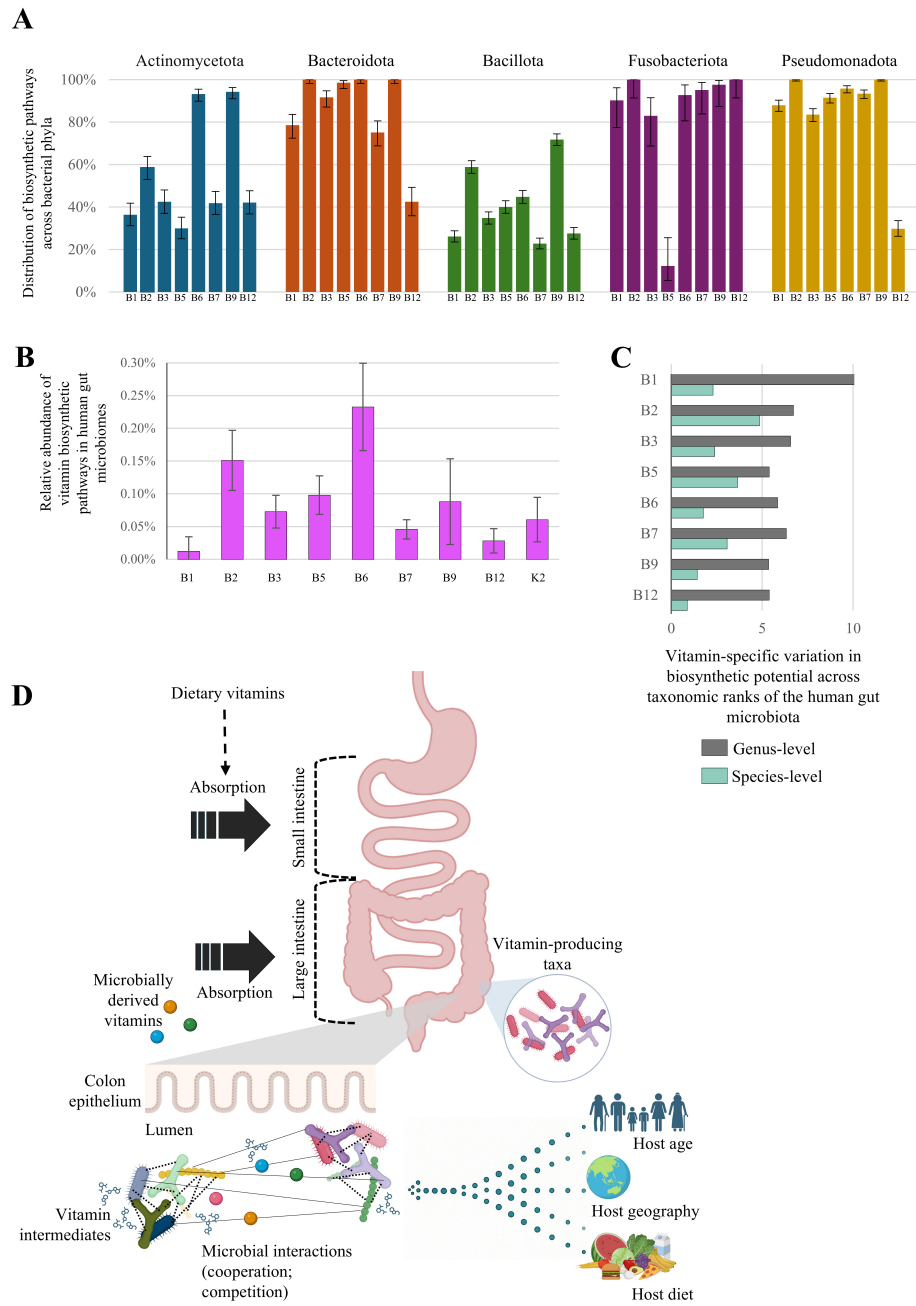
Gut microbiota as a source of B-group vitamins and vitamin K. (A) Occurrence of de novo biosynthetic pathways for B-group vitamins across major gut bacterial phyla, assessed from > 2,000 reference genomes. Error bars indicate 95% Wilson confidence intervals; (B) Relative abundance of individual vitamin biosynthetic pathways in the human gut microbiota, based on > 4,000 adult gut metagenomes. Error bars represent the interquartile range (IQR); (C) Vitamin-specific variability scores across microbial taxa within each taxonomic rank, reflecting diversity in biosynthetic potential, calculated as described by Rodionov *et al.*^[37]^; (D) Schematic illustrating absorption of microbially derived B vitamins in the human colon Vitamin availability is shaped by microbe-microbe interactions (competition and cooperation) and host factors such as age, diet, and geographic origin. Created in BioRender. Mancabelli, L. (2025).

Recent genomic investigations conducted on a curated reference collection of microbial genomes have enabled a detailed characterization of the vitamin biosynthetic potential across individual gut microbiota members^[29,37]^. When these insights are summarized at the phylum level, members of the phyla Bacteroidota and Pseudomonadota appear enriched in vitamin prototrophic species (organisms capable of synthesizing a particular compound *de novo*) [Figure 1A]. These phyla include species able to synthesize all B vitamins, except cobalamin, whose biosynthetic potential was found in only 30% of Bacteroidota and 42% of Pseudomonadota genomes. In contrast, Actinomycetota and Bacillota were shown to include a higher proportion of vitamin auxotrophic members, i.e., species that depend on exogenous sources to meet their vitamin requirements^[29,37]^ [Figure 1A]. More specifically, only some members of the genus *Bacteroides* possess the metabolic potential to synthesize all eight B-group vitamins^[29,37]^. Alongside *Bacteroides* spp., other major contributors to B vitamin production in the human gut include *E. coli*, *Phocaeicola dorei*, members of the *Bifidobacterium* genus and *Segatella copri* (previously *Prevotella copri*)^[22]^. Interestingly, these genera are also hallmarks of distinct gut microbiome configurations known as enterotypes or gut community state types^[38]^, which, because of differences in microbial composition, have been shown to differ in their overall repertoire of B vitamin biosynthetic abilities^[38]^. Specifically, enterotype 1, typically dominated by members of the genus *Bacteroides*, is enriched in genes involved in biotin, riboflavin, and pantothenic acid biosynthesis, whereas enterotype 2, characterized by a predominance of *Segatella copri*, displays a higher representation of genes associated with thiamine and folate biosynthesis^[38]^. In contrast to these prototrophic species, various gut microbiota members are auxotrophic for B vitamins and thus rely on external sources to meet their metabolic needs. For instance, the strictly anaerobe *Faecalibacterium prausnitzii* is considered a major consumer of several B vitamins, including riboflavin, which is required for its survival in the moderately oxygenated gut environments by acting as an extracellular antioxidant.

While genomic studies have elucidated the biosynthetic capacities of individual strains or genomes, metagenomic approaches have extended this perspective to entire microbial communities, allowing the mapping of vitamin biosynthetic potential at the population level^[22,39]^. For instance, in the gut microbiota of individuals aged 18 to 70 years, microbial genes involved in the biosynthesis of B-group and K2 vitamins collectively accounted for approximately 1%-2% of the total metagenome, with particularly high contributions from vitamins B2 and B6 [Figure 1B]^[22]^.

Together, these findings highlight the pivotal role of specific microbial taxa in shaping the gut vitamin landscape through a pattern of biosynthetic capacities and vitamin-utilization strategies. A deeper understanding of taxon-specific contributions to vitamin metabolism is crucial for elucidating the functional interplay between the microbiota and host nutritional status. Such insights may inform the design of microbiome-based interventions aimed at optimizing vitamin availability and addressing nutrient deficiencies.

In this context, the recognized ability of lactobacilli from various fermented foods to secrete folate has already found practical application in the development of starter cultures for folate-fortified dairy products. In parallel, strains formerly classified within the genus *Lactobacillus* and isolated from the human gut have been explored as candidate probiotics for their folate-producing potential^[40,41]^. These examples illustrate how knowledge regarding microbial vitamin metabolism can be translated into both food-based and therapeutic strategies, ultimately paving the way for nutritional approaches that exploit the beneficial metabolic potential of the gut microbiome.

### Microbe-microbe and microbe-host interactions in vitamin availability

While the vitamin biosynthetic ability of the human gut microbiota is increasingly recognized, recent findings have highlighted that vitamin availability within the gut microbiota is not solely determined by biosynthetic capacity, but also by complex metabolic interactions within the microbial community and at the host interface. These dynamics include microbe-microbe cross-metabolism, competition for vitamin uptake, and host-mediated absorption, all of which shape the nutritional output.

At a microbial level, vitamin cross-feeding metabolism arises from the heterogeneous distribution of biosynthetic capabilities among taxa, which can occur not only between distant phylogenetic groups but also among species within the same genus^[37,42]^. In a recent study, a manually curated reference collection of more than 2,000 genomes from human gut-associated bacteria was analyzed using the SEED platform^[43]^, which integrates all known and inferred components of biosynthetic and salvage pathways, including enzymes and transporters. This approach enabled a quantitative assessment of the variability in vitamin biosynthetic potential by predicting the proportion of prototrophic and auxotrophic strains across different taxonomic ranks^[37]^ [Figure 1C]. This genomic analysis revealed that phenotypic variability increases progressively at higher taxonomic levels and enabled the identification of biosynthetic pathways that are unevenly distributed even among closely related taxa^[37]^. Notably, riboflavin biosynthetic capabilities showed the greatest strain-level variability, whereas cobalamin biosynthesis emerged as the most conserved at the genus level [Figure 1C]. In contrast, thiamine biosynthesis exhibited the highest degree of variability at both genus and family levels, suggesting a broader evolutionary flexibility in this pathway^[37]^ [Figure 1C].

These patterns of conservation and variability across vitamins and taxonomic levels provide a foundation for potential metabolic interactions within microbial communities. Indeed, when prototrophic and auxotrophic microorganisms co-occur within the same ecological niche, this disparity can rise to metabolic interdependence, whereby metabolic products or intermediates released by prototrophs support the growth and metabolic functions of auxotrophic counterparts^[44,45]^. A clear example is vitamin B12, a structurally complex and metabolically expensive cofactor^[20]^. Due to the high metabolic cost and genetic burden associated with maintaining its complete biosynthetic pathway, only a limited subset of anaerobic gut bacteria, primarily within the Bacillota and Actinomycetota phyla, are able to autonomously produce vitamin B12. Nevertheless, this cofactor is essential for numerous community members, many of whom have evolved high-affinity transport systems to acquire it from their environment. For example, *Anaerobutyricum hallii* (formerly *Eubacterium hallii*), a well-characterized butyrate producer, is capable of synthesizing vitamin B12, which is symbiotically utilized by *Akkermansia* species for propionate biosynthesis^[46]^. Similarly, in synthetic gut microbial community models, growth of *Roseburia intestinalis* M50/1, which is a strain auxotrophic for folate, has been shown to benefit from the presence of *Bifidobacterium bifidum* CNCM I-3650, an *in vitro* validated folate prototroph model^[47]^. These findings have suggested that prototrophic bacteria can alleviate auxotrophic limitations of co-occurring strains, either through active metabolite/micronutrient exchange or via passive mechanisms such as cell lysis and subsequent release of intracellular vitamins.

These vitamin-mediated interactions are not merely cooperative; competition also plays a critical role in shaping vitamin availability in the human gut^[48,49]^ [Figure 1D]. Microbes compete for limited vitamin pools by producing vitamin-binding proteins and employing selective transporters to outcompete other community members^[50]^. Accordingly, the gut environment can be viewed as a tightly regulated micronutrient marketplace, where availability is shaped by both biosynthetic capacity and uptake efficiency. For example, *Bacteroides thetaiotaomicron* expresses three functional, homologous vitamin B12 transporter systems for cobalamin acquisition. These transporters exhibit distinct specificities for different corrinoid analogs, likely conferring a selective advantage in the competitive human gut environment^[51]^, and illustrating the sophisticated strategies and extensive efforts employed by gut microbes to secure essential cofactors^[51]^.

Host-microbe interactions further complicate this network. Indeed, the host can indirectly shape vitamin dynamics through immune regulation, colonic absorption, mucus production, and diet, all of which influence microbial composition and function. Consistently, it has recently been shown that host-associated factors, such as age and geographic origin, significantly influence the vitamin biosynthetic potential of the human gut microbiota^[22]^ [Figure 1D]. Specifically, shotgun metagenomic-based analysis has shown that, during infancy, biosynthetic pathways for vitamin B9 and K2 synthesis are highly prevalent and abundant, consistent with the dominance of *Bifidobacterium* spp., which are generally capable of synthesizing folate, and *E. coli*, predicted to produce K2, in the early-life gut microbiome^[22]^. Another study, by stratifying the population into age groups ranging from a few months to over 80 years and correlating microbial features with age, revealed that the neonatal group (0-4 years) showed a positive correlation with enzymes involved in the biosynthesis of thiamine and niacin, suggesting a specific association of these vitamin pathways with early life, in contrast to older age groups^[52]^. Conversely, the adult gut microbiota, enriched in species belonging to the *Bacteroides* genus and *Segatella copri* (previously *Prevotella copri*), exhibits an expanded metabolic repertoire, as these taxa are generally B vitamin prototrophs equipped with complete biosynthetic pathways for *de novo* biosynthesis of multiple B vitamins^[22,29,44]^. Furthermore, emerging evidence indicates that geographic origin contributes to differences in the gut microbiota capacity for B vitamin biosynthesis across global populations. Notably, adults from the United States exhibit a pronounced and widespread depletion of gut microbiome-derived B vitamin biosynthetic pathways, compared with populations from eastern regions of the globe, likely due to diet- and lifestyle-associated factors that are presumed to affect gut microbiota composition and functionality^[22]^.

Within this complex ecological and metabolic network, elucidating which microbial taxa contribute to vitamin biosynthesis in the colon, whether these vitamins are absorbed by the host, and which factors influence their production is essential to clarify the nutritional contribution of the microbiota to the host, as well as to design microbiota-targeted strategies to enhance vitamin status through prebiotics, probiotics, or engineered consortia.

## DISSECTION OF MICROBIAL SYNTHESIS OF VITAMINS IN THE HUMAN GUT

In the following section, we outline the microbial biosynthesis of each B-group vitamin and vitamin K2, summarizing their physiological relevance and the current knowledge on the microbial taxa and biosynthetic pathways involved.

### Vitamin B1 (Thiamine)

#### Biological role and importance

Thiamine (vitamin B1) is a water-soluble vitamin required as a coenzyme in several central metabolic processes. In its active phosphorylated form, TPP, vitamin B1 serves as a critical cofactor for multiple enzymes involved in carbohydrate metabolism, including pyruvate dehydrogenase, α-ketoglutarate dehydrogenase, and transketolase, which are key players in the tricarboxylic acid cycle (TCA) and the pentose phosphate pathway^[53-57]^. Through these pathways, thiamine contributes to energy generation, redox balance, biosynthesis of nucleotides and lipids, and indirectly to neurotransmitter metabolism^[57]^.

#### Microbial biosynthesis and known microbial contributors

Thiamine biosynthesis in bacteria follows a modular pathway that involves the separate synthesis of two moieties: 4-amino-5-hydroxymethyl-2-methylpyrimidine phosphate (HMP-P) and 4-methyl-5-(β-hydroxyethyl)thiazole phosphate (THZ-P). These are then coupled by the enzyme ThiE to produce thiamine phosphate, which is subsequently converted to the active TPP form^[56]^. Genomic surveys have indicated that a substantial proportion of gut-associated bacterial genomes, including representatives of major genera such as *Bacteroides*, *Prevotella*, *Enterococcus*, and *Bifidobacterium*, harbor the complete genetic repertoire required for the *de novo* biosynthesis of thiamine phosphate^[29,37,58]^.

Nonetheless, many gut commensals are auxotrophic for thiamine, relying on exogenous thiamine or intermediates such as HMP-P, which can be salvaged from the environment or exchanged between microbes^[58]^. Among these vitamin B1 auxotrophs, different salvage-based strategies have been identified: while some strains can recover both thiamine intermediates, others lack one biosynthetic branch and rely on external sources for the corresponding missing compound, and a subset depends entirely on the uptake of pre-formed thiamine. Notably, all auxotrophic variants retain some salvage capacity, reflecting diverse evolutionary adaptations to incomplete biosynthetic potential^[37]^.

Interestingly, this vitamin is required by *F. prausnitzii* for the metabolic conversion of pyruvate into acetyl-CoA as part of the butyrate biosynthesis pathway, underscoring its pivotal role in short-chain fatty acid (SCFA) production by members of the gut microbiota.

### Vitamin B2 (Riboflavin)

#### Biological role and importance

Riboflavin (vitamin B2) acts as a precursor to two major flavocoenzymes: FMN and FAD, which are essential for a wide range of redox reactions in central metabolism, including those involved in the electron transport chain, fatty acid oxidation, and amino acid catabolism, and the activation of vitamin B6 and vitamin B9^[59,60]^. Riboflavin also contributes to the maintenance of antioxidant defenses by supporting glutathione reductase activity and regulating oxidative stress^[60,61]^.

#### Microbial biosynthesis and known contributors

Riboflavin biosynthesis in bacteria involves a conserved set of genes (*rib*A, *rib*B, *rib*C, *rib*D, and *rib*E) that convert guanosine 5' -triphosphate (GTP) and ribulose-5-phosphate into riboflavin, which is then phosphorylated to FMN and further adenylated to FAD. Recent genome-scale reconstructions highlight that the complete operon is widely distributed among commensal bacteria in the gut, including *Bacteroides*, *Enterococcus*, and several lactobacilli^[21,29,37]^. Notably, *Lactococcus lactis*, *Lactiplantibacillus plantarum*, and *Limosilactobacillus reuteri* have been proposed as natural bio-enriching agents for increasing riboflavin concentration in various food matrices, such as yoghurt, cheeses, and fermented milk products^[62-65]^. However, genome-based analyses have also revealed that the complete riboflavin biosynthetic operon is absent in several members of the Bacillota and Actinomycetota phyla^[23]^. Interestingly, many of these riboflavin-auxotrophic organisms encode high-affinity riboflavin transport systems, such as *Rib*U (ECF family) and *Rib*XY (ABC superfamily), suggesting a strong physiological demand for riboflavin-derived cofactors, including FMN and FAD.

Beyond its nutritional role, microbially produced riboflavin has also been implicated in modulating host immune responses at mucosal surfaces. The key mechanism involves mucosal-associated invariant T (MAIT) cells, which are activated upon recognition of riboflavin biosynthesis intermediates presented by the non-polymorphic MHC class I-related molecule MR1^[66]^. This pathway establishes a direct link between microbial vitamin B2 metabolism and mucosal immune surveillance^[67-69]^, underscoring the broader physiological relevance of microbial riboflavin production in host-microbe interactions.

### Vitamin B3 (Niacin)

#### Biological role and importance

Vitamin B3, or niacin, encompasses two biologically active forms, represented by nicotinic acid (NA) and nicotinamide (NAM), both of which serve as precursors to nicotinamide adenine dinucleotide (NAD^+^) and nicotinamide adenine dinucleotide phosphate (NADP^+^). These dinucleotides are essential cofactors involved in redox reactions, energy metabolism, DNA repair, and cellular signaling^[70,71]^. NAD^+^ functions as a key coenzyme in glycolysis, the TCA cycle, and oxidative phosphorylation, whereas NADP^+^ is crucial for anabolic pathways and antioxidant defense^[72]^.

#### Microbial biosynthesis and known contributors

Unlike most other B-group vitamins, niacin can be synthesized endogenously in humans, primarily in the liver, via the kynurenine pathway that converts tryptophan into quinolinate, a key intermediate in NAD^+^ biosynthesis^[73]^. However, this route is generally considered insufficient to meet daily requirements, making dietary intake or microbial contribution particularly important^[73]^. In the gut, many commensal bacteria possess an alternative *de novo* biosynthetic pathway for niacin, which starts from aspartate and leads to the formation of quinolinate, subsequently converted into NA mononucleotide and ultimately into NAD^+^^[74]^. This biosynthetic pathway is widely distributed among Pseudomonadota and Bacteroidota, although significant variability exists at the genus and species level^[29,37]^. Notably, *E. coli*, *B. fragilis*, and various *Bifidobacterium* species have been identified as niacin producers in the gut environment^[22,29,37]^. However, not all species encode the full set of biosynthetic genes, and many rely on niacin salvage pathways, which enable them to utilize host- or microbe-derived precursors^[37]^. Recent evidence highlights a bidirectional exchange of vitamin B3 precursors between the host and its gut microbiota, supporting a shared NAD^+^ metabolism^[75]^. While niacin precursors from the diet are mostly absorbed in the upper gut/small intestine, microbes in the colon can synthesize NAD^+^ from fermentable fibers such as inulin, or from host-derived NAM released through cellular metabolism. Microbially derived NAD⁺ is subsequently converted into NAM and NA, which can be absorbed by host intestinal tissues and used to regenerate NAD^+^ within host cells^[75-77]^ [Supplementary Figure 1]. This highlights a dynamic cycle in which both microbial activity and host-derived substrates contribute to maintaining circulating levels of NA, even in the absence of direct dietary intake^[37]^.

### Vitamin B5 (Pantothenic Acid)

#### Biological role and importance

Pantothenic acid is the biochemical precursor of CoA, which is a central metabolic cofactor involved in a wide array of biological processes^[78]^. CoA plays a pivotal role in the TCA cycle, fatty acid synthesis and β-oxidation, and amino acid metabolism, acting as an acyl group carrier in energy-yielding and anabolic pathways^[78,79]^. It is also required for the biosynthesis of cholesterol, steroid hormones, heme, and acetylcholine^[80]^. Given its broad involvement in intermediary metabolism, B5 is vital for maintaining cellular energy balance and metabolic functions.

#### Microbial biosynthesis and known contributors

Pantothenic acid is synthesized *de novo* in bacteria via a pathway that combines pantoate with β-alanine. The pathway is encoded by a highly conserved gene cluster (*pan*B, *pan*C, *pan*D, *pan*E), and the resulting vitamin is then converted to CoA^[79]^.

In the human gut microbiota, genomic analyses have shown that nearly all Pseudomonadota and Bacteroidota possess the genetic capacity to synthesize pantothenate, whereas less than half of the assessed Bacillota and Actinomycetota members encode a complete pantothenate pathway^[29,37]^. Nevertheless, many pantothenate auxotrophs retain the enzymatic machinery necessary to convert exogenous pantothenate into CoA, enabling them to fulfill essential metabolic functions even in the absence of de novo synthesis^[29,37]^. Reflecting the widespread biosynthetic competence observed in Pseudomonadota and Bacteroidota, biosynthetic pathways for vitamin B5 biosynthesis rank among the most represented in the human gut microbiota during adulthood, particularly within the archetypal *Bacteroides*-dominated enterotype^[22,38]^.

### Vitamin B6 (Pyridoxine)

#### Biological role and importance

Vitamin B6 refers to a group of six interconvertible vitamers: pyridoxine (PN), pyridoxal (PL), pyridoxamine (PM), and their respective phosphorylated forms, of which pyridoxal 5’-phosphate (PLP) is the most biologically active^[81]^. PLP acts as a coenzyme in approximately 140 enzymatic reactions cataloged by the Enzyme Commission (EC; http://www.chem.qmul.ac.uk/iubmb/enzyme/), making vitamin B6 one of the most functionally versatile and complex micronutrients^[81,82]^. Except for glycogen phosphorylases, nearly all PLP-dependent enzymes are involved in biochemical processes related to amino compounds, particularly amino acid metabolism^[81]^.

#### Microbial biosynthesis and known contributors

In the human gut, PLP is synthesized via two distinct pathways: the longer deoxyxylulose 5-phosphate (DXP)-dependent pathway, prevalent in Bacteroidota and Pseudomonadota, and the shorter DXP-independent pathway, more common in Actinomycetota^[29,83]^.

Genomic analyses have demonstrated that PLP production is not uniformly distributed across all gut bacteria^[29,37]^. While microbial genera such as *Bifidobacterium*, *Bacteroides*, and *Prevotella* are predicted to produce PLP, other prominent gut commensals, such as multiple *Veillonella* spp., *Enterococcus faecalis*, *F. prausnitzii*, and *Roseburia inulinivorans*, have been predicted to be auxotrophic. Importantly, these organisms often carry genetic signatures for salvage pathways, enabling them to convert environmental B6 vitamers into usable PLP^[37]^.

### Vitamin B7 (Biotin)

#### Biological role and importance

Biotin, also known as vitamin H, is a sulfur-containing water-soluble vitamin that functions as a coenzyme for carboxylase enzymes involved in crucial metabolic pathways, including fatty acid metabolism, amino acid metabolism, carbohydrate metabolism, polyketide biosynthesis, and urea utilization^[84]^. Covalently bound to target enzymes via a lysine residue, biotin facilitates carbon dioxide transfer reactions essential for maintaining energy homeostasis and lipid metabolism^[85]^.

#### Microbial biosynthesis and known contributors

Biotin biosynthesis in bacteria proceeds through two distinct pathways. In *Escherichia coli*, the biotin molecule is synthesized via a modified fatty acid synthesis pathway that requires the *bio*C and *bio*H genes^[86]^. This route is considered the canonical and most widespread among biotin-producing gut bacteria. In contrast, *Bacillus subtilis* utilizes a separate pathway, centered around the *bio*W gene. This *bio*W-dependent route appears to be more common among members of the Bacillota phylum^[29]^. According to recent genomic investigations, all analyzed genomes from the phylum Actinomycetota lacked the essential genes required for *de novo* biotin synthesis, a feature that was also infrequently observed among Bacillota^[29,37]^. Consistent with their auxotrophic profile, the majority of Actinomycetota encoded a high-affinity biotin uptake system^[37]^. This finding underscores their reliance on environmental biotin sources and suggests the existence of selective mechanisms to ensure efficient scavenging of this essential cofactor.

### Vitamin B9 (Folate)

#### Biological role and importance

Vitamin B9 comprises a family of water-soluble compounds structurally related to THF, which functions as a coenzyme in one-carbon metabolism^[87-89]^. THF and its derivatives are essential for the transfer of one-carbon units in reactions involved in the synthesis of purines, thymidylate, and several amino acids, participating also in nucleotide synthesis and replication, repair, and methylation of DNA^[88,89]^. Folate is therefore fundamental for nucleotide synthesis, epigenetic regulation, and cell division, making it particularly critical during periods of rapid growth such as pregnancy and infancy.

#### Microbial biosynthesis and known contributors

*De novo* folate biosynthesis in bacteria requires the production and subsequent condensation of two key intermediates: dihydroneopterin triphosphate (DHPPP) and para-aminobenzoic acid (pABA)^[90]^. These precursors are synthesized through distinct metabolic routes and ultimately converge to form dihydrofolate (DHF), which is then reduced to THF, the biologically active form of folate^[90]^.

Among gut microbes, infant gut-specialized species including *B. bifidum*, *B. longum*, and *B. breve* have been recognized as key folate producers^[15,40,91]^. These strains have demonstrated the ability to accumulate folate in the culture medium^[91,92]^, suggesting a potential contribution to the folate pool in the infant gut^[22]^. However, it is important to note that bifidobacteria generally require exogenous pABA supplementation to enable folate biosynthesis, indicating a partial auxotrophy for this precursor^[15]^.

Lactobacilli represent another bacterial folate-producing group^[40]^. Specifically, strains belonging to species such as *Lactobacillus delbrueckii*, *Lactobacillus helveticus*, *Limosilactobacillus reuteri*, *Latilactobacillus sakei*, and *Lactiplantibacillus plantarum* have been reported to synthesize folate when pABA is available in the growth environment. Notably, *L. plantarum* constitutes an exception among lactobacilli, as it shows a consistent capacity to produce folate without pABA supplementation^[93,94]^.

Interestingly, many studies assessed the contribution of intestinal microbiota to the folate intake of animal hosts^[95-97]^, and it has been demonstrated that the folate synthesized by intestinal bacteria can be absorbed in the colon and used by the host^[96,98,99]^. Building on this observation, lactobacilli from various fermented foods have been investigated as starter cultures for the manufacture of folate-fortified dairy products, while lactobacilli isolated from the human gut have been explored as folate-producing probiotics^[100,101]^.

### Vitamin B12 (Cobalamin)

#### Biological role and importance

Vitamin B12, or cobalamin, is a cobalt-containing cofactor and one of the largest and most structurally complex nonpolymeric biomolecules found in nature^[102]^. In humans, it acts as a coenzyme in two essential mitochondrial reactions: methylmalonyl-CoA mutase, which participates in propionate metabolism and energy production, and methionine synthase, which plays a key role in the regeneration of methionine from homocysteine in the folate cycle^[102]^.

Among its various forms, cyanocobalamin is the most chemically stable form^[102]^; upon absorption, it undergoes enzymatic processing and is converted into its active cofactor forms, represented by methylcobalamin (Me-Cbl) and adenosylcobalamin (Ado-Cbl)^[102,103]^.

#### Microbial biosynthesis and known contributors

Cobalamin biosynthesis is a complex and energetically demanding process that involves more than 30 genes, following either an aerobic or anaerobic pathway, both converging in the formation of the corrin ring and final cobalt chelation^[20,104]^. These pathways are largely restricted to specific bacterial taxa, being notably absent in many gut microbes. Indeed, recent large-scale genomic analyses have revealed that approximately 60%-80% of the assessed human gut commensals lack the complete gene repertoire required for cobalamin production, including members of the *Bifidobacterium* and *Prevotella* genera, as well as *Bacteroides thetaiotaomicron*^[37,105]^. In contrast, *Propionibacterium freudenreichii*, *Clostridium* spp., *Bacteroides fragilis*, *Akkermansia muciniphila*, and some Bacillota and Actinomycetota encode complete or nearly complete B12 biosynthetic operons^[37,105]^. However, due to the considerable energetic demands associated with B12 biosynthesis, numerous bacteria have evolved alternative strategies to secure this cofactor and its analogs (corrinoids) from the surrounding environment^[20,106]^. These include salvage of precursors or uptake of the fully formed molecule from other community members, fostering cooperative interactions and resource-sharing dynamics within the gut microbiome^[19,51]^.

Nevertheless, in humans, colonic B12 is not absorbed efficiently, making the direct contribution of microbiota-derived B12 to systemic levels uncertain^[107]^. Additionally, only a subset of microbial cobalamin analogs is bioactive in humans^[19,30]^, raising concerns about the functional compatibility between host requirements and microbial products^[19]^. Some microbial corrinoids can bind to host receptors, yet do not activate B12-dependent enzymes, further questioning their biological relevance in shaping host cobalamin status.

### Vitamin K2 (Menaquinones)

#### Biological role and importance

Vitamin K exists in two primary forms: phylloquinone (K1), found in green leafy vegetables, and menaquinones (K2), a diverse group of isoprenoid derivatives produced primarily by bacteria. Menaquinones (MK-n) differ in the length of their isoprenoid side chain, which affects their bioavailability and tissue distribution^[108]^. In bacterial cells, MK-n play a central role in electron transport across the cytoplasmic membrane, support sporulation processes, and contribute to the virulence of pathogenic bacteria^[109]^. In humans, inadequate vitamin K2 levels have been associated with osteoporosis, cardiovascular disease, and impaired insulin sensitivity^[110]^.

#### Microbial biosynthesis and known contributors

The menaquinone biosynthesis pathway in bacteria involves the convergence of two crucial components, involving the naphthoquinone ring and the isoprenyl side chain^[111]^. These two components are first produced via distinct metabolic routes and subsequently condensed to form the quinone core structure^[112-114]^. The isoprenoid side chain then undergoes elongation through sequential additions of isopentenyl units, resulting in menaquinone variants of different chain lengths^[115]^. This structural diversity underpins functional specialization and may contribute to the environmental adaptability of different bacterial species^[111,116]^.

Several menaquinone-producing bacterial strains are currently used as starters in industrial food fermentation, demonstrating the ability to enrich the growth medium with vitamin K. For example, when *Lactococcus cremoris*, *L. lactis*, and *Leuconostoc lactis* have been grown in reconstituted dry milk or soy milk, these strains produced long-chain MKs in concentrations ranging from 29 to 123 μg/L^[115,117]^, representing amounts approaching the daily adequate intake recommended for adults (55-120 μg/day)^[118]^. These findings support the idea that fermented foods can serve as a significant dietary source of vitamin K2.

## METHODOLOGIES FOR STUDYING THE MICROBIAL VITAMIN BIOSYNTHETIC NETWORK

In recent years, major advances in high-throughput sequencing, multi-omics, and computational modeling have dramatically expanded our understanding of the human gut microbiome, including its engagement in vitamin biosynthesis^[119]^. In this section of the review, we outline the diverse set of complementary methodologies that can be deployed to investigate microbial vitamin metabolism. These include metagenomic and genomic approaches to map biosynthetic potential; analytical chemistry techniques to detect and quantify vitamins and their derivatives; transcriptomic and metabolomic tools to assess gene expression and enzymatic activity; and functional assays in both isolated cultures and complex microbial communities. Furthermore, *in vivo* systems, including germ-free animal models and human intervention studies, are essential to establish the physiological relevance of microbial vitamin production and exchange.

Crucially, the integration of these approaches, within a multi-omics framework, offers a powerful means to link genotype to phenotype, predict metabolic interactions, and ultimately reconstruct the structure and function of the vitamin biosynthetic network within the gut microbiota. As the field moves forward, interdisciplinary collaboration across microbiology, computational biology, analytical chemistry, and systems biology will be key to resolving the remaining uncertainties and unlocking the therapeutic potential of microbial vitamin metabolism.

### Metagenomic and bioinformatic approaches to mapping community-level vitamin biosynthetic potential

**Figure 2:**
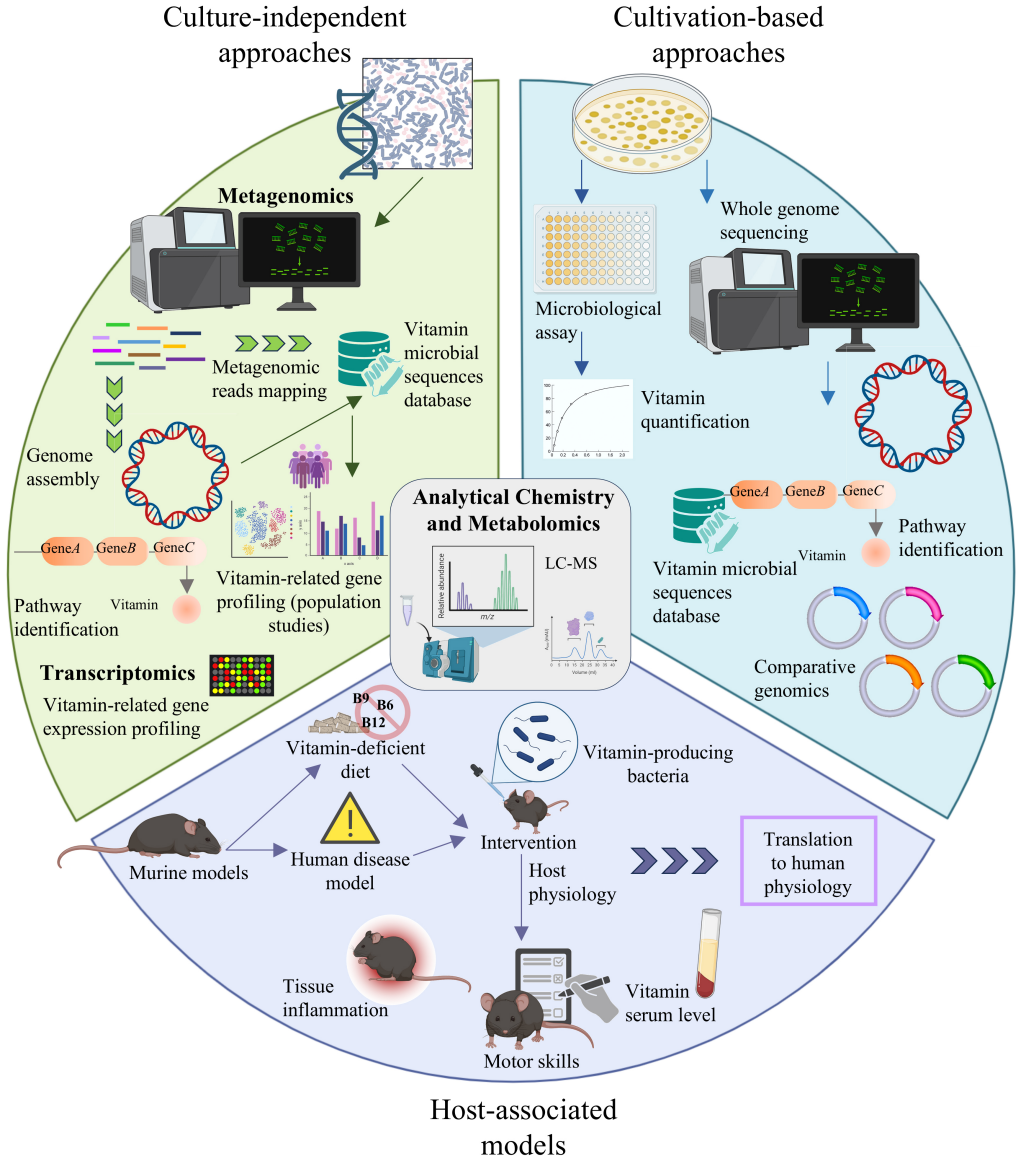
Schematic overview of the key methodological approaches used to assess microbial vitamin biosynthesis, interspecies interactions, and physiological relevance *in vivo*. The diagram illustrates culture-independent methods (metagenomics, transcriptomics), culture-dependent strategies (isolation, genome sequencing, microbiological assays), and host-associated models (e.g., murine systems). Analytical chemistry and metabolomics methods can complement and be integrated across all these approaches to directly detect and quantify vitamin production and exchange.

Metagenomics represents a foundational tool for dissecting the metabolic potential of microbial communities, offering a genome-wide, cultivation-independent snapshot of gene content across all microbial taxa present in a sample. When applied to the study of vitamin biosynthesis, metagenomic sequencing has enabled the identification of biosynthetic genes and transporters involved in vitamin metabolism across diverse gut environments in host populations [Figure 2].

A key strength of metagenomics lies in its ability to profile both abundant and rare community members, enabling detection of vitamin biosynthetic potential even in low-abundant taxa that may serve as key metabolic contributors. Functional annotation of metagenomic reads or contigs against curated databases, such as KEGG (https://www.genome.jp/kegg/), MetaCyc (https://metacyc.org/), Rhea^[120]^, SEED (http://www.theseed.org/), and Protein Data Bank (PDB, https://www.wwpdb.org/), may reveal the presence and completeness of biosynthetic pathways for B-group vitamins and menaquinones^[22,39]^. Annotation can be performed using tools such as HUMAnN3^[121]^, METAnnotatorX2^[122]^, and MG-RAST^[123]^, which employ BLAST-like tools such as DIAMOND^[124]^, as well as the read aligners Bowtie2 and BWA^[125,126]^, to map gene content to functional modules, enabling reconstruction of community-level metabolic potential. In parallel, detection of vitamin transporter-encoding genes provides additional evidence of auxotrophy or cross-feeding capabilities within the community.

Metagenomic analyses can be performed using two principal approaches: assembly-based and assembly-free [Figure 2]. In the assembly-based strategy, reads are assembled into longer contigs or bins, often with the goal of recovering metagenome-assembled genomes (MAGs), which represent reconstructed genome sequences of microbial populations present in the sample^[127]^. This enables the attribution of vitamin biosynthetic genes to specific taxa and supports genome-scale modeling of the metabolic potential of recovered microorganisms^[128]^. However, assembly is computationally intensive and may suffer from fragmentation or bias against low-abundance organisms.

In contrast, assembly-free approaches rely on read-level annotation, providing rapid community-level estimates of functional potential. While less precise, this method has been used to compute large-scale screening across populations, diets, or disease states^[22,39]^. For example, recent studies have shown that individuals consuming high-fiber diets display gut microbiomes enriched in folate- and riboflavin-producing taxa, while Western-style diets are associated with greater auxotrophy and reduced biosynthetic capacity^[22]^. Distinct patterns of microbial vitamin metabolism in the gut have also been linked to host age, with adults generally exhibiting greater biosynthetic capacity, and infants showing a relative enrichment in menaquinone and folate production^[22]^. In clinical cohorts, impaired abundance of vitamin K2 and various B vitamin biosynthesis genes has been observed in type 2 diabetes patients, suggesting a link between microbial vitamin metabolism and host health status^[39,129]^.

Despite these advances, (meta) genomic inference is inherently limited to potential function, as it does not capture gene expression or enzymatic activity. Moreover, current databases may lack annotations for alternative pathways or recently discovered enzymes, especially for non-model organisms. Therefore, predictions drawn from metagenomic data must be interpreted cautiously and ideally integrated with complementary functional and experimental data. In this context, community transcriptomics represents a robust strategy for uncovering gene expression dynamics along entire biosynthetic pathways^[130]^. A recent study, for instance, demonstrated that the expression of genes involved in cobalamin and thiamine biosynthesis was significantly reduced in inflammatory bowel disease (IBD) patients compared to healthy controls, highlighting the functional impact of disease on microbial vitamin metabolism^[39]^. However, studies applying transcriptomics, either as a standalone approach or as part of multi-omics frameworks, are still scarce, highlighting a promising yet underexplored area of microbiome research.

Overall, metagenomic and bioinformatic approaches provide a powerful entry point for mapping the genetic potential of gut microbes to synthesize, transform, or acquire vitamins. While they cannot resolve functional output on their own, they may guide the design of targeted experiments, identify candidate producers, and model metabolic interactions at the community level.

### Genomic approaches to mapping the vitamin biosynthetic potential

In parallel with community-level investigations, whole-genome sequencing (WGS) offers a powerful approach to characterize the genetic potential of individual bacterial strains^[131]^, including the complete repertoire of vitamin biosynthetic pathways [Figure 2]. The decreasing cost of DNA sequencing and the widespread adoption of this technology have resulted in a vast and growing repository of publicly available microbial genomes, allowing researchers to access and scrutinize a broad range of taxa without the need for laboratory cultivation^[132]^ [Figure 2].

WGS of bacterial isolates, or the analysis of publicly available genome sequences, offers a high-resolution view of the genetic architecture underlying vitamin biosynthesis at the strain level. The process typically begins with the generation of high-quality genome assemblies through short-read (e.g., Illumina) or long-read (e.g., Oxford Nanopore, PacBio) sequencing technologies. The assemblies are then annotated either with classic approaches, such as Prodigal^[133]^, followed by homology-based functional annotations, or modern platforms, such as Prokka^[134]^ or RAST^[135]^, which perform comprehensive gene annotation pipelines. Gene content is then mapped to curated databases such as KEGG, MetaCyc, and Rhea, to assess pathway completeness and to infer metabolic capabilities or dependencies. Recent studies have successfully applied these approaches to identify genes encoding enzymes involved in vitamin biosynthetic pathways, as well as those related to vitamin transport and salvage systems^[29,37,106]^.

By extending this strategy to a broad set of phylogenetically diverse microorganisms, comparative genomic analyses have revealed substantial heterogeneity in vitamin production capacity across microbial lineages, even within the same species, allowing for identification of candidate vitamin producers and the prediction of interspecies cross-feeding networks within the gut microbiota^[37,44]^ [Figure 2]. For example, while genes involved in riboflavin and niacin biosynthesis are widely distributed among gut microbes, complete cobalamin biosynthetic pathways are relatively uncommon, occurring in only a minority of vitamin B12-requiring organisms^[106]^. This observation aligns with broader predictions from *in silico* reconstructions, which suggest that a significant portion of the human gut microbial community, often exceeding 20% in relative abundance, is composed of auxotrophic species unable to synthesize one or more B vitamins^[44]^. These taxa instead depend on dietary sources or on cross-feeding interactions with prototrophic members capable of vitamin production, underscoring the ecological significance of vitamin exchange and vitamin-mediated nutritional competition in shaping microbial community structure and function.

Together, these findings highlight the value of genomics in elucidating the metabolic roles of individual strains and their contributions to vitamin dynamics in microbial communities.

### Cultivation-based approaches for studying microbial vitamin synthesis

While sequencing-based methods have greatly expanded our understanding of microbial metabolism, culture-based approaches remain essential for experimentally validating vitamin biosynthesis in microbes. Cultivation allows for direct testing of metabolic capabilities, the isolation of vitamin producers and auxotrophs, and the design of controlled experiments to investigate microbial interactions under defined nutritional conditions.

A classical and still widely used method to assess vitamin biosynthetic potential in laboratory conditions is the use of auxotrophic indicator strains, whose growth depends on the external availability of specific B-group vitamins, enabling quantification based on growth response^[136-138]^ [Figure 2]. Such assays have been applied extensively to study folate, thiamine, and cobalamin production in lactic acid bacteria and bifidobacteria^[91,139]^.

*In vitro* systems have also been employed to directly observe microbial vitamin cross-feeding by pairing auxotrophic and prototrophic isolates. For instance, in a folate-depleted medium, *R. intestinalis* was able to grow when co-cultured with *B. bifidum*, which passively releases folate intermediates into the environment^[47]^. Similarly, *Roseburia faecis* sustained the growth of *F. prausnitzii*, auxotrophs for thiamin, in the absence of exogenous sources of this micronutrient, indicating effective inter-species nutrient sharing^[47]^.

To better replicate the complexity of gut microbial ecosystems and extend the duration of observation, more sophisticated *in vitro* culturing platforms such as bioreactor-based fermentation systems have been employed^[140,141]^. These closed, dynamic systems enable the controlled modulation of environmental parameters (e.g., pH, retention time, substrate input), while supporting stable and reproducible “synthetic” microbial communities over time^[142-144]^. Although such systems have been extensively used to investigate microbial ecology and metabolite production under simulated colonic conditions^[141,145]^, their direct application to the study of B vitamin biosynthesis and exchange by gut microbes remains relatively limited. Nevertheless, they offer a promising framework for future studies aimed at capturing microbial nutrient interactions under more physiologically relevant conditions.

Despite these advancements, cultivation-based approaches face inherent limitations. A substantial proportion of gut microbes are not yet culturable under standard laboratory conditions, restricting our ability to assess vitamin metabolism of key community members^[146]^. Nonetheless, cultivation remains a cornerstone of functional microbiology, enabling direct experimental validation of vitamin biosynthesis, transport, and interspecies exchange^[47,147]^.

### Analytical chemistry methods and metabolomics

Within the “omics” technologies, metabolomics plays a central role in elucidating microbial metabolism, offering direct evidence through the detection and quantification of metabolite production^[148,149]^. Unlike genomics or transcriptomics, which infer metabolic potential, metabolomics captures the actual outcome of microbial metabolism, thus serving as a powerful tool to probe host- and microbe-microbe metabolic interactions, including micronutrient dynamics^[150,151]^.

Metabolomics approaches are broadly categorized into targeted and non-targeted strategies^[152]^. Targeted metabolomics focuses on the precise quantification of specific metabolites, such as B vitamins or their derivatives/vitamers, using known standards^[153]^. In contrast, non-targeted metabolomics aims to provide a global overview of the metabolome, enabling the identification of unexpected or novel metabolic changes across different biological systems^[154]^. At present, our ability to detect, characterize, and quantify metabolites relies on the rapid advancement of a wide range of analytical chemistry platforms, including gas chromatography (GC), liquid chromatography (LC), and high- and ultra-performance LC (HPLC, UPLC)^[155,156]^. Among these, LC, particularly when coupled with mass spectrometry (LC-MS), has emerged as one of the most versatile and widely used tools in metabolomics^[157]^. Typically coupled with electrospray ionization (ESI), LC-MS enables the simultaneous analysis of multiple B vitamins and menaquinones in complex matrices, such as foods, milk, pharmaceutical formulations, infant formulas, blood, and human feces^[158-160]^.

An advanced variant of this approach, liquid chromatography-tandem mass spectrometry (LC-MS/MS), further enhances sensitivity and specificity by employing two sequential stages of mass analysis^[161]^. This configuration not only enables the detection of trace-level compounds but also incorporates the ability to distinguish structurally similar molecules, such as isoforms of the same vitamin. For instance, LC-MS/MS has recently been applied to achieve the simultaneous and precise quantification of three active forms of vitamin B12, demonstrating its value in the fine-resolution analysis of micronutrient diversity.

Although these analytical strategies have been successfully employed to distinguish and quantify active forms of various B-group vitamins from complex food and biological matrices, their application to the study of microbial vitamin biosynthesis in the human gut remains limited. Nonetheless, their high specificity and sensitivity make them a promising avenue for future research aimed at elucidating microbially derived vitamin dynamics *in vivo*.

### Preclinical models

Understanding the contribution of gut microbial vitamin biosynthesis to host physiology requires models that integrate microbial activity with host responses. While *in vitro* studies and multi-omics approaches provide essential mechanistic insights, host-associated models are indispensable for assessing the physiological relevance, bioavailability, and systemic effects of microbially derived vitamins.

Among the most widely adopted systems used to study host-microbiota interactions are animal/rodent models, particularly mice and rats, which provide a controlled environment to investigate microbiota-dependent processes [Figure 2]. These models are valuable not only for examining nutritional influences, such as the restoration of folate levels in folate-deficient rats by folate-overproducing strains of *Bifidobacterium adolescentis* and *B. pseudocatenulatum*^[98]^, but also for simulating human disease contexts [Figure 2]. Given the essential role of B vitamins in various human disorders, murine models have proven instrumental in exploring the impact of microbial vitamin metabolism on host pathophysiology.

In this regard, vitamin-synthesizing probiotic strains have shown promise in treating inflammatory diseases, especially those affecting the gastrointestinal tract^[162-165]^. For instance, the riboflavin-producing *Lactiplantibacillus plantarum* CRL2130 strain effectively suppressed inflammatory cytokines and alleviated symptoms in mouse models of ulcerative colitis and intestinal mucositis^[166,167]^. Similarly, *Streptococcus thermophilus* strains CRL808 and CRL415, which produce folate, reduced (chemically induced) mucosal inflammation in murine models^[168]^. Moreover, when administered together, folate- and riboflavin-producing strains have been shown to enhance chemotherapy efficacy in breast cancer models while minimizing side effects such as mucositis^[168]^.

B vitamin-producing strains have also demonstrated neuroprotective effects. In murine models of Parkinson’s disease, oral administration of either the riboflavin-producing *L. plantarum* CRL2130 or the thiamine-producing *L. plantarum* CRL1905 improved motor coordination and prevented dopaminergic neuronal loss^[169,170]^. These effects, accompanied by reduced systemic and brain-localized inflammation, were comparable to those of commercial vitamin supplementations, confirming that microbial vitamin production is bioavailable to and functionally relevant for the host, supporting host nutritional needs and contributing to physiological processes.

Despite their value, host-associated models also come with inherent limitations. Rats and mice differ in anatomy, immunity, and metabolism from humans, and the interpretation and translation of findings require caution^[171,172]^. Nevertheless, these models remain essential for bridging the gap between *in vitro* predictions and human physiology, offering a crucial platform to validate functional hypotheses in a whole-organism context.

In summary, host-associated models are indispensable tools for demonstrating the functional relevance of microbial vitamin metabolism. They enable researchers to explore host-microbiota interactions *in vivo*, quantify microbial contributions to vitamin homeostasis, and design interventions to modulate these interactions for improved health outcomes. Nevertheless, despite the evidence from murine models, clinical data directly addressing the role of microbial vitamins or vitamin-producing bacterial strains in human health remain scarce. Bridging this gap will require well-designed clinical trials that specifically assess the efficacy of vitamin-producing strains, a step that is essential to translate findings from animal models into therapeutic strategies for humans.

## CONCLUSIONS AND FUTURE PERSPECTIVES

The intricate web of microbial vitamin biosynthesis in the human gut is emerging as a central aspect of microbiome-host interactions. Rather than merely reflecting a simple metabolic function, the ability of gut microbes to synthesize, share, and modulate vitamin availability represents a strategic axis of ecological cooperation, host support, and evolutionary adaptation^[23]^.

During the last decade, advances in high-throughput sequencing, functional genomics, and integrative omics have revealed that many gut bacteria do not possess complete biosynthetic routes for essential vitamins. Instead, they engage in metabolic cross-feeding, forming cooperative networks in which intermediates or fully formed vitamins are exchanged among community members^[37,44]^. These networks provide a compelling example of how the microbiota operates not just as a collection of individual organisms, but as a cooperative metabolic entity, which in turn is intimately tied to host wellbeing.

A comprehensive understanding of this ecosystem-level metabolism requires a multidisciplinary methodological arsenal. Genomics and metagenomics enable the mapping of biosynthetic potential, while transcriptomics and metabolomics reveal functional activity and regulation^[173,174]^. Cultivation and experimental validation provide causative evidence and mechanistic insights, whereas animal models are essential for validating microbial contributions in physiological contexts. Although studies in disease models have shown that certain lactic acid bacteria (LAB), including specific strains of *Lactiplantibacillus plantarum*, *Streptococcus thermophilus* species, and members of the *Bifidobacterium* genus, exert protective effects associated with their vitamin-producing abilities, their efficacy in humans has yet to be demonstrated. Translating these findings into clinical benefit requires overcoming several challenges, such as ensuring strain stability during production and gastrointestinal transit, establishing effective and standardized dosages, and accounting for host- and diet-dependent variability in vitamin biosynthesis. Addressing these issues through carefully designed human studies will be essential to substantiate the therapeutic potential of vitamin-producing probiotics.

Only through the integration of these approaches can we capture the full depth and nuance of microbial vitamin metabolism.

Looking forward, several challenges and opportunities remain. First, we must improve the resolution and functional interpretation of ‘omics’ data, especially for non-model microbial taxa and non-canonical vitamin variants. This requires more complete and curated databases, and high-resolution omics to refine our predictions and uncover the regulatory and ecological dynamics of vitamin metabolism.

Importantly, there is a growing need to translate basic discoveries into practical interventions. By using probiotics with defined vitamin production profiles, tailoring diets to stimulate microbial biosynthesis, or developing personalized microbiome-based nutritional therapies, we may develop novel strategies for nutritional support, disease prevention, and personalized health care.

In conclusion, dissecting the gut microbiota vitamin biosynthetic network is not only a fascinating scientific field of research, but also a promising path toward harnessing the full metabolic potential of our microbial partners for lifelong health.

## DECLARATIONS

### Copyright

© The Author(s) 2025.
