# Supplementary material for: Approaches to dissect the vitamin biosynthetic network of the gut microbiota
Source: Microbiome Res Rep. 2025 Oct 20;4(4):37. doi: 10.20517/mrr.2025.66 (PMC12702654; doi:10.20517/mrr.2025.66)
Supplement: Supplementary file 1 [file mrr-4-4-37-SupplementaryMaterials.pdf]

Supplementary Materials

Approaches to dissect the vitamin biosynthetic network of the gut microbiota

Chiara Tarracchini[1], Francesca Bottacini[2,3], Leonardo Mancabelli[4,5], Gabriele Andrea Lugli[1,5], Francesca Turroni[1,5], Douwe van Sinderen[2,6], Marco Ventura[1,5], Christian Milani[1,5]

[1]Laboratory of Probiogenomics, Dept. Chemistry, Life Sciences and Environmental Sustainability, University of Parma, Parma 43124, Italy.
[2]APC Microbiome Ireland, University College Cork, Cork T12 YN60, Ireland.
[3]Department of Biological Sciences, Munster Technological University, Cork T12 P928, Ireland.
[4]Department of Medicine and Surgery, University of Parma, Parma 43124, Italy.
[5]Microbiome Research Hub, University of Parma, Parma 43124, Italy.
[6]School of Microbiology, Bioscience Institute, National University of Ireland, Cork T12 Y337, Ireland.

Correspondence to: Christian Milani, Laboratory of Probiogenomics, Department of Chemistry, Life Sciences, and Environmental Sustainability, University of Parma, Parma 43124, Italy. E-mail: christian.milani@unipr.it; Marco Ventura, Laboratory of Probiogenomics, Department of Chemistry, Life Sciences, and Environmental Sustainability, University of Parma, Parma 43124, Italy. E-mail: marco.ventura@unipr.it

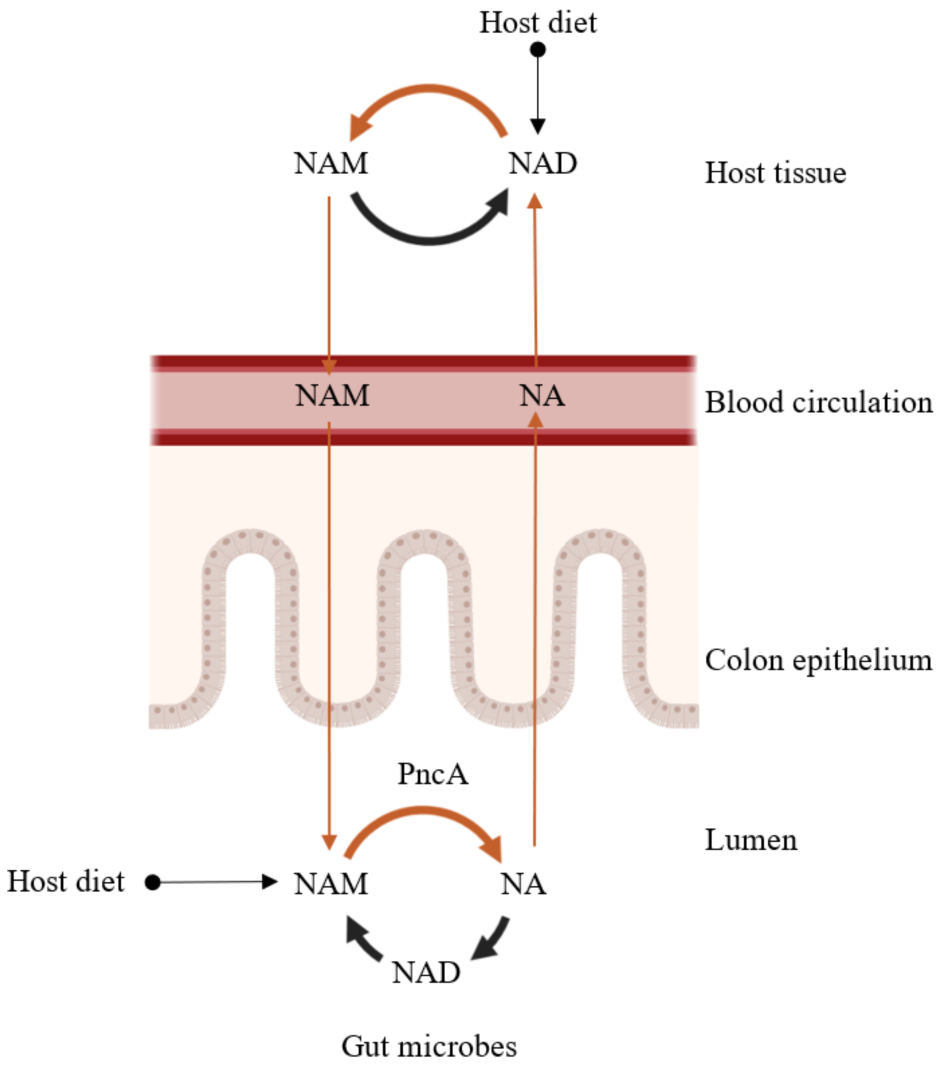

Supplementary Figure 1. Simplified schematic of the vitamin B3 cycle between host and microbiota. Host-derived nicotinamide (NAM) enters the gut lumen and is converted by gut bacteria into nicotinic acid (NA). Microbiome-produced NA is then utilized by host tissues for nicotinamide adenine dinucleotide (NAD) synthesis, which, upon turnover, releases NAM back into circulation. Created in BioRender. Mancabelli, L. (2025).

REFERENCES

Shats I, Williams JG, Liu J, et al. Bacteria Boost Mammalian Host NAD Metabolism by Engaging the Deamidated Biosynthesis Pathway. Cell Metab. 2020;31(3):564-579.e7. https://doi.org/10.1016/j.cmet.2020.02.001

Feng, S., Guo, L., Wang, H. et al. Bacterial PncA improves diet-induced NAFLD in mice by enabling the transition from nicotinamide to nicotinic acid. Commun Biol 6, 235 (2023). https://doi.org/10.1038/s42003-023-04613-8
